# Supplementary material for: Saccharomyces boulardii (CNCM I-745) improves intestinal damage in sepsis by remodeling intestinal flora structure
Source: Front Cell Infect Microbiol. 2025 Jul 31;15:1602792. doi: 10.3389/fcimb.2025.1602792 (PMC12350329; doi:10.3389/fcimb.2025.1602792)
Supplement: Supplementary file 1 [file DataSheet1.zip › summary/summary/7_Differential_analysis/2_LEfSe_result/Group_BW4_vs_AW4_0.05_3.0/LEfSe_Group_BW4_vs_AW4.s.barplot.pdf]

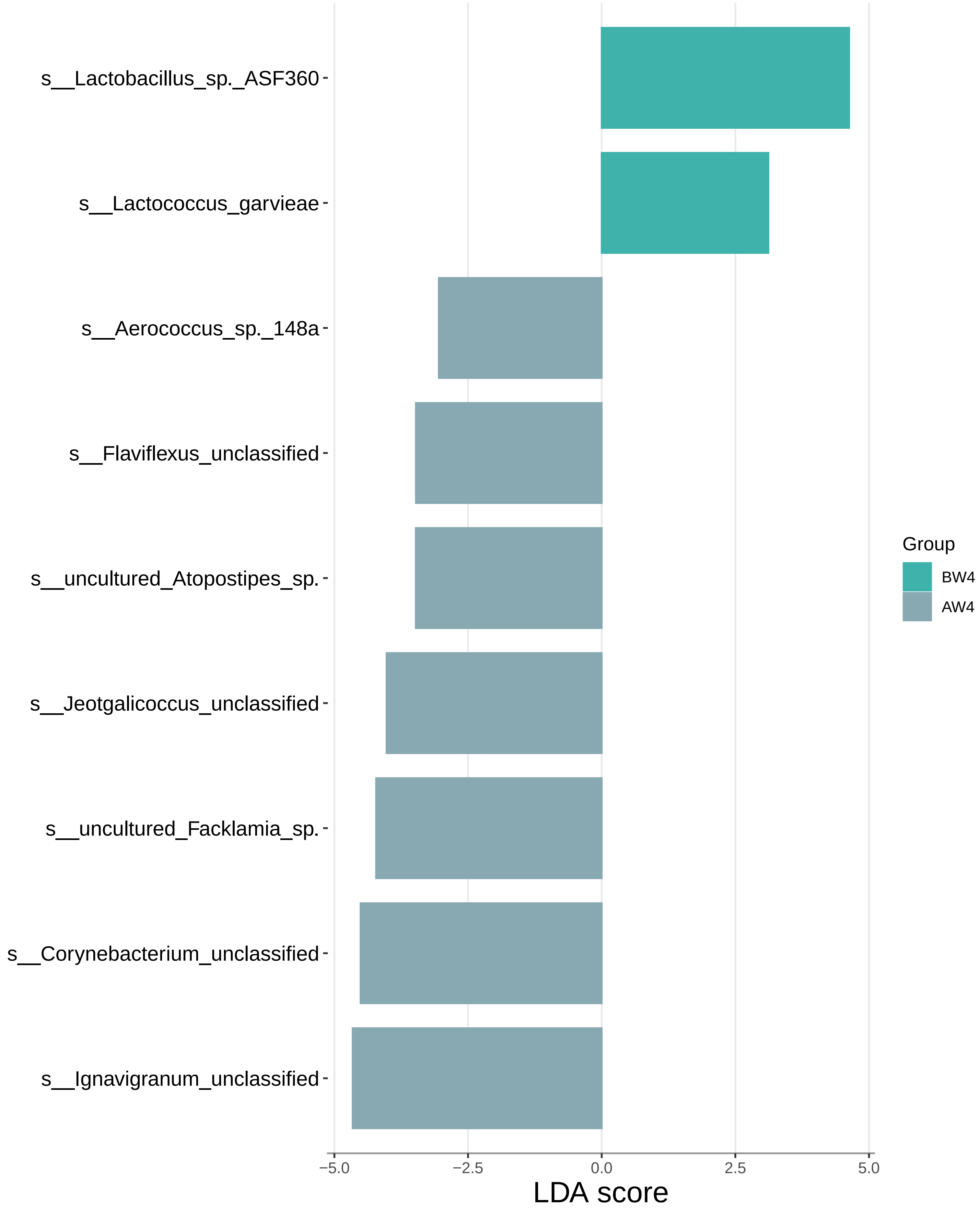
s__Lactobacillus_sp._ASF360
s__Lactococcus_garvieae
s__Aerococcus_sp._148a
s__Flaviflexus_unclassified
s__uncultured_Atopostipes_sp.
s__Jeotgalicoccus_unclassified
s__uncultured_Facklamia_sp.
s__Corynebacterium_unclassified
s__Ignavigranum_unclassified
−5.0
−2.5
0.0
2.5
5.0
LDA score
Group
BW4
AW4
